# On the Influence of Vaccination in Diminishing the Mortality from Small-Pox

**Published:** 1847-02

**Authors:** 


*On the Influence of Vaccination in Diminishing the Mortality from Small-Pox.*—Much has recently been said respecting the frequency and fatality of small-pox, at the same time that doubts were often expressed as to the afficacy of vaccination in preventing that virulent disease. Should any person still entertain such opinions, his fears must be very considerably diminished by the facts stated at page 62 in the Seventh Report of the Registrar-General, just published. According to that official document, the number of deaths from small-pox, throughout England and Wales, during five consecutive years, were as follows:—In 1838, 16,268 persons died of that disease; in 1839, 9,131; in 1840, 10,434; in 1841, 6,368; and in 1842, only 2,715 deaths are reported.—*Ibid.*

